# From Heat Stroke to Multi-Organ Failure: A Survivor's Case Report

**DOI:** 10.7759/cureus.48984

**Published:** 2023-11-18

**Authors:** Hanna Elbashir, Leena Saeed, Doaa Sabir, Marwa Morgom, Yara Abuazab, Tasneem Madebo, Alhady A Yusof

**Affiliations:** 1 Emergency Medicine, Hamad General Hospital, Doha, QAT; 2 Internal Medicine, Hamad Medical Corporation, Doha, QAT; 3 Medicine and Surgery, Jordan University of Science and Technology, Irbid, JOR; 4 Medicine and Surgery, National Ribat University, Khartoum, SDN; 5 Critical Care, Hamad Medical Corporation, Doha, QAT

**Keywords:** critical care, multiple organ dysfunction syndrome, heat related, multi-organ failure, heat stroke

## Abstract

A heat stroke (HS) is a medical emergency that can occur when the body is unable to cool itself down after overexertion in a hot condition. It is characterized by a high body temperature (usually greater than 40.5 degrees Celsius or 104.9 degrees Fahrenheit) and altered mental status. HS can cause a wide range of physiological changes in the body, including damage to the brain, heart, liver, kidneys, and muscles. In the case report presented, the patient was a 40-year-old man who developed severe HS. His condition rapidly deteriorated, and he developed multi-organ failure, involving the brain, liver, kidneys, muscles, and hematological system. The patient was admitted to the intensive care unit (ICU) and intubated, despite aggressive treatment. After an 18-day stay in the ICU, the patient achieved full recovery except for myopathy, which necessitated physiotherapy.

## Introduction

Heat-related illness (HRI) is a range of conditions that vary in severity, from heat exhaustion to heat stroke (HS). These conditions are caused by the body's inability to regulate its temperature, which can result in extreme hyperthermia, central nervous system dysfunction, and multi-organ failure. HRI can occur when the body fails to dissipate heat during extremely hot weather or when heat production exceeds the body's ability to dissipate it during physical activity, particularly among young, active adults and athletes [1-3].

HS is the most severe form of HRI. It can lead to serious complications, such as multi-organ dysfunction syndrome, rhabdomyolysis, acute renal failure, coagulopathy, acute respiratory distress syndrome (ARDS), hepatic dysfunction, and myocardial injury [1].

We report a case of multi-organ failure secondary to heat stroke in a 40-year-old gentleman. It shows how timely intervention can reduce the incidence of death and disability associated with heat stroke.

## Case presentation

A 40-year-old male patient with no past medical history was brought to the emergency department (ED) after being found unresponsive in the street, febrile with bulging eyes bilaterally and few abrasions on his face. When paramedics arrived, he was unresponsive with a temperature of 40 degrees Celsius. He was intubated at the scene and transported immediately to the ED for further evaluation. An initial evaluation in the ED revealed an intubated patient with a Glasgow coma scale of 3/15 (E1VTM1), temperature of 42 degrees, tachycardic with a heart rate of 190 beats per minute, respiratory rate of 24 breaths per minute, blood pressure of 110/57 mmHg maintained with noradrenaline, and oxygen saturation was 98% on continuous mechanical ventilation requiring 100% FiO_2_. He was continuously passing watery diarrhea at the time of resuscitation in the ED due to a loss of sphincter control.

On examination, the patient was shivering; the eye examination showed bilateral exophthalmos with pinpoint pupils not reacting to light; the chest was clear with normal air entry bilaterally and no added sounds; the abdomen was soft and lax; and there was no lower limb edema. Bedside focus assessment with sonography for trauma (FAST) was negative, and cardiac point of care ultrasound showed an ejection fraction of 40% and a collapsed inferior vena cava (IVC). Arterial line, central line, and esophageal temperature probe were inserted for accurate and continuous measurements and management. Immediate cooling maneuvers were started in the ED using ice packs, cooling blankets, and cold generous IV fluids. As the patient was passing watery diarrhea continuously, a fecal catheter was inserted. Within one hour in the ED, his temperature subsequently reached 36 degrees Celsius, his FiO_2_ requirement decreased to 50% and PEEP 7, heart rate of 130 bpm, and blood pressure of 84/65 mmHg, which is then maintained with the increase in vasopressor rate. The patient was shifted to an intensive care unit for close monitoring.

**Figure 1:**
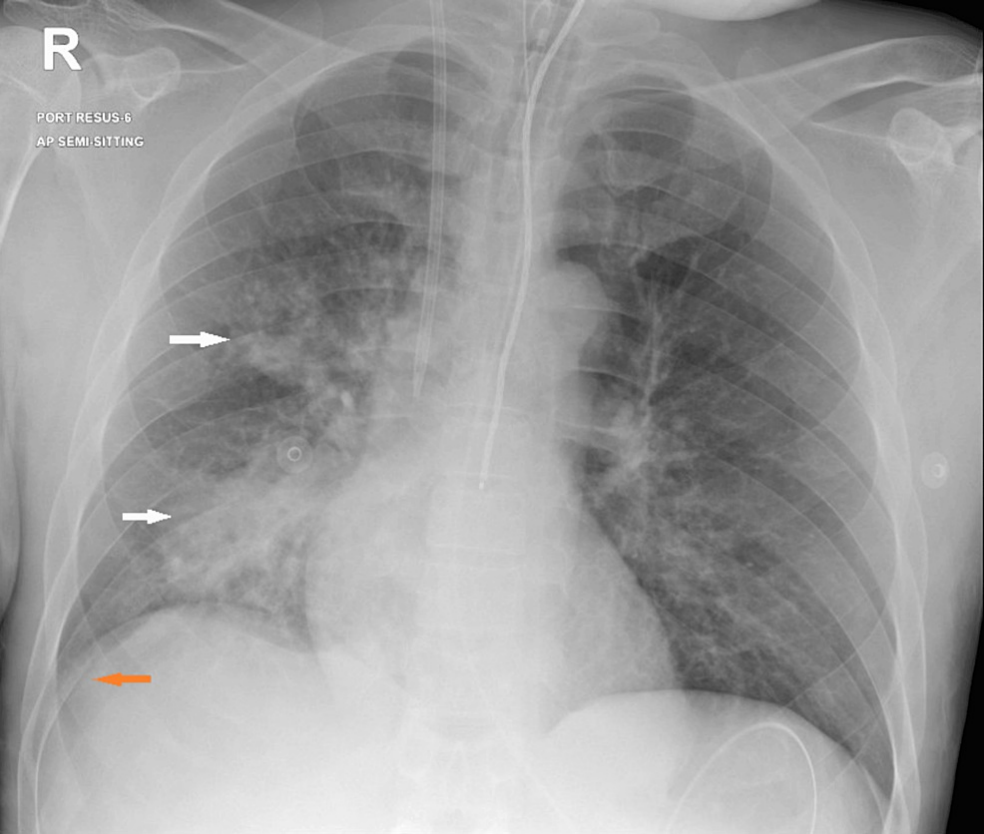
Interval increase in right mid- and lower lung zone patchy opacities (white arrow) and mild pleural effusion (orange arrow).

Initial labs and cultures were drawn (see Table 1), and a CT scan of the head and a chest x-ray were unremarkable. Repeated chest x-ray after three hours revealed an interval increase in right mid- and lower lung zone patchy opacities and mild pleural effusion (Figure 1). Therefore, started on Augmentin 1 g IV empirically and then escalated to a piperacillin/tazobactam 4500 mg infusion. However, after *Pseudomonas aeruginosa* and *Enterobacter cloacae* growth from lower respiratory secretion culture, the antibiotic was changed to cefepime 1 g infusion according to the sensitivity and considering his acute renal impairment.

**Table 1:** Pertinent lab values summary. WBC: white blood cell; Hb: hemoglobin; PT: prothrombin time; INR: international normalized ratio; aPTT: activated partial thromboplastin time; AST: aspartate aminotransferase; ALT: alanine aminotransferase; ALP: alkaline phosphatase; T.Bi.: total bilirubin; LDH: lactate dehydrogenase; CK: creatine kinase; CRP: c-reactive protein; TSH: thyroid stimulating hormone; fT4: free T4.

	Initial labs	8-hrs	20-hrs	70-hrs	18 Days (day of ICU step down)	Reference values
WBC	10.0	4.4	9.6	10.9	9.6	4-10 (10 x 10^3^/uL)
Eosinophils	0.00					0.02-0.50 (10 x 10^3^/uL)
Hb	12.7	12.4	10.3	8.7	8.7	13-17 (gm/dl)
Platelets	106	12	34	36	454	150-410 (x10^3^/uL)
PT	12.6	19.8	18.8	16.9	14.1	9.4-12.5 (seconds)
INR	1.1	1.7	1.6	1.4	1.3	0.9-1.1
APTT	28	29.9	34.3	30.2	29.7	25.1-36.5 (seconds)
Fibrinogen	1.63	1.70				2-4.1 (gm/L)
D-dimer	6.15	5.30			2.97	0.00-0.44 (mg/L)
Peripheral smear	Mild normochromic normocytic anemia with anisocytosis. There is no increase in schistocytes. Thrombocytopenia.					
Sodium	128		136	140	132	133-146 (mmol/L)
Potassium	3.1		3.8	3.3	3.9	3.5-5.3 (mmol/L)
Bicarb	11		22	26	21.7	22-29 (mmol/L)
Urea	5.9		9.4	9.3	4.50	2.5-7.8 (mmol/L)
Creatinine	146		210	270	70	62-106 (umol/L)
AST	1381		529	248	82	0-40 (U/L)
ALT	244		139	93	39	0-41 (U/L)
ALP	84		35	67	67	40-129 (U/L)
T. Bi.	9		10	22	7.2	0-21 (umol/L)
LDH	1327					135-225 (U/L)
CK	1426			3854	600	39-308 (mmol/L)
Lactic acid	4.70	2.40	4.6	1.30	0.6	0.5-2.2 (mmol/L)
Amylase	58					13-53 (U/L)
Lipase	339					13-60 (U/L)
Procalcitonin		7.51			0.09	<0.05 (ng/mL)
CRP	1.2		19.8	139.4	13	
Troponin	800	1286	603		76.2	3-15 (ng/L)
Myoglobin	3820		1909	1134	161	0-85 (pg/ml)
TSH	4.53					0.30-4.20 (mlU/L)
fT4	10.8					11-23.3 (pmol/L)
Acetaminophen	21					Therapeutic range 66-199 (umol/L)
Salicylate	<0.3					15-30 (mg/dL)
Ethanol	<2.2					Critical high >44.9 (mmol/L)
Urine toxicology screen	Negative					
Urinalysis	Negative for infection					
Blood culture	No growth					
Urine and stool culture	No growth					
Respiratory lower culture				Positive (*Pseudomonas aeruginosa*/*Enterobacter cloacae*)		

Supportive care was provided with maintenance intravenous fluid infusions, mechanical ventilator support, nutritional support, electrolyte correction, and close monitoring. The electrocardiogram showed only sinus tachycardia. An echocardiogram performed during intubated status revealed an ejection fraction of 40%, mildly reduced LV systolic function, mild global hypokinesis of the LV, and grade 1 diastolic dysfunction.

Despite improvement in his temperature and hemodynamics, he started to deteriorate in terms of rapid worsening of liver enzymes, worsening kidney function, rhabdomyolysis, thrombocytopenia, and coagulopathy (Table 1). He had mild bleeding from the mouth and nose. He was given a 500 mg injection of tranexamic acid and had a fibrinogen and platelet concentrate transfusion due to low fibrinogen levels of 1.70 g/L and platelets of 12 x 10^3^/uL. He was given an acetylcysteine infusion of 150 mg/kg for his liver injury. A diagnosis of heat stroke was made as the etiology of his acute kidney injury (AKI), acute liver injury, and coagulopathy.

After 12 days of his stay in the ICU, the patient retained his full consciousness. Although he weaned from mechanical ventilation, he was still requiring oxygen support through a high nasal flow cannula. He had stepped down to the general ward on day 18. His general condition was stable, but he reported having general body weakness, which is secondary to post-critical illness myopathy. Therefore, he was transferred to a rehabilitation center to continue physiotherapy.

## Discussion

HS is a severe form of heat-related illness, commonly manifested by the presence of high body temperature of equal or greater than 40.5 degrees Celsius, abnormalities in the central nervous system, occurrence of bleeding and coagulation disorders as well as multi-organ damage. It can be classified into two types: (a) classical (non-exertional) HS, which is most common in children, the elderly, and people with chronic illnesses, and (b) exertional HS, which usually affects physically active individuals such as army members or those who are performing strenuous exercises [4,5]. In our case, the patient was found unresponsive in the street; therefore, the exact history of whether he was practicing any physical activity in the sun or not, is unknown.

Early recognition of heat stroke is crucial for prompt management and stabilization of the patient. Additionally, it is important to rule out other potential diagnoses such as thyroid storm, sepsis, or neurological causes like meningitis by doing thyroid function test, sepsis workup, and conducting brain imaging. Treatment for HS involves rapidly cooling the body temperature within the golden hour of treatment. This can be done by immersing the patient in cold water, pouring water on the body and fanning, infusing cold IV fluids, or applying ice packs or wet gauze sheets. Respiratory support by providing oxygen, hyperbaric oxygenations, and mechanical ventilation is crucially important, as well as the provision of intensive care unit management. In addition, nutritional support and electrolyte correction play a core role in management. Dialysis and liver transplants may be required in some cases of kidney and liver dysfunction [4,6,7].

In the event of delayed improvement in the temperature by usually used methods, it is imperative to explore alternative cooling methods such as immersion of the patient in an ice-cold bath, low-temperature hemodialysis, and bladder irrigation with cold fluids.

Early intubation performed by prehospital paramedics, followed by prompt transfer to the hospital, early identification and evaluation of alternative diagnoses, and swift implementation of continuous monitoring probes such as arterial line, central line, and esophageal temperature probe were crucial components in the management of our patient. Additionally, expedited resuscitative measures to address elevated body temperature, utilization of adjuncts such as bedside US, ECHO, and IVC assessment, as well as monitoring of end-tidal carbon dioxide (ETCO2) levels. Moreover, the importance of arterial line waveform morphology, which plays a vital role in guiding fluid administration and predicting fluid responsiveness during rapid fluid therapy. Furthermore, the incorporation of adjuncts like fecal catheter for continuous diarrhea can significantly enhance the overall management process.

Our patient's condition is complicated by multiple organ dysfunction syndrome (MODS). Studies have shown that complications of HS are happening due to the presence of high levels of inflammatory cytokines, direct injury to the body organs, endothelium, and microvascular circulation, and the decrease of blood flow to the core organs. Subsequently, systemic inflammatory response syndrome (SIRS) and MODS will occur [4,8]. Therefore, as a result of these abnormalities, patients develop acute kidney injury, liver damage, disseminated intravascular coagulopathy (DIC), and rhabdomyolysis.

It's been reported that the mortality rate of ICU-admitted patients who develop rhabdomyolysis and AKI may reach up to 59% [4,8]. It is evident in our case clinical condition and his labs that the occurrence of these syndromes has led to serious outcomes and prolonged ICU admission. Patients don't usually recover in most published cases [9-11]. However, in our case, the patient was able to survive after prompt treatment and interventions.

## Conclusions

To sum it up, HS is a severe form of heat-related illness and it can present with a wide spectrum of manifestations of organ damage. Commonly reported complications include AKI, liver injury, DIC, rhabdomyolysis, multi-organ failure, and death. Recovery is uncommon in the majority of reported cases. However, in our case, the patient was able to survive. Upon illustrating HS management, we highlight that early prehospital intervention by paramedics and transportation to the hospital, early recognition of the symptoms and management with enough hydration and cooling as well as close monitoring and supportive care are the key points of the treatment. 
